# Evaluation of Vision-related Quality of Life in Patients with Glaucoma: A Hospital-based Study

**DOI:** 10.5005/jp-journals-10078-1242

**Published:** 2019

**Authors:** Munish Dhawan, Tania Hans, Pawandeep S Sandhu, Neha Midha

**Affiliations:** 1–3 Department of Ophthalmology, Guru Gobind Singh Medical College and Hospital, Faridkot, Punjab, India; 4 Dr Rajendra Prasad Centre for Ophthalmic Sciences, All India Institute of Medical Sciences, New Delhi, India

**Keywords:** Glaucoma, GQL 15 questionnaire, Quality of life

## Abstract

**Introduction:**

Quality of life (QoL) is a broad concept that is affected in a complex way by many factors. Healthcare interventions are targeted now days to improve quality of life of affected individuals. Glaucoma is a major cause of irreversible blindness throughout the world and affects patient's quality of life in several ways. In present study, we aim to evaluate and quantify vision related quality of life in glaucoma patients in a tertiary care hospital setting.

**Materials and methods:**

Vision related quality of life was evaluated in glaucoma patients using GQL-15 questionnaire, which compares the subjective performance of various vision related tasks in these patients. Fifty diagnosed cases of glaucoma and fifty healthy volunteers were included in the study. In both these groups, standard tests for visual function were done and both were assigned to complete the questionnaire. GQL-15 questionnaire includes 15 items divided between 4 factors pertaining to visual disability: central and near vision, peripheral vision, dark adaptation and glare, and outdoor mobility. Higher scores indicate greater difficulty in performing vision-related activities and poorer QoL.

**Results:**

A total of 100 cases were enrolled out of which 50 were diagnosed glaucoma cases and 50 were controls. Almost three fourth of glaucoma patients, i.e., 72% were diagnosed as chronic open angle glaucoma where 24% were angle closure patients and rest 4% were normal tension glaucoma patients. The mean GQL score of glaucoma cases was 26.00 ± 10.84 and for controls it was 15.02 ± 0.14 (*p* value < 0.05). All subscale scores also showed a uniform rise in their value as we move from mild to severe cases thereby concluding that all visual parameters worsen with increase in severity pattern of disease.

**Conclusions:**

As glaucoma patients have reduced vision related quality of life, so every effort should be made to preserve visual functions in these patients. Many activities that define independence and productivity in society require good vision and hence one of most devastating consequences of advancing visual impairment in glaucoma is progressive loss of independence thereby affecting patients quality of life.

**How to cite this article:**

Dhawan M, Hans T, *et al.* Evaluation of Vision-related Quality of Life in Patients with Glaucoma: A Hospital-based Study. J Curr Glaucoma Pract 2019;13(1):9–15.

## INTRODUCTION

The term quality of life (QoL) is defined by the World Health Organization as the subjective perception of well-being and wholeness.^1^ It is a broad concept that is affected in a complex way by many factors. QoL measures are increasingly recognized as important outcomes in understanding the impact of a disease and evaluating the effectiveness of healthcare interventions.^2^ Hence, it is important to evaluate the relative importance of symptoms or disabilities to better understand the effect of a disease in a patient's QoL.

Glaucoma is a heterogeneous group of diseases that have in common a characteristic form of damage to the optic nerve head. The damage generally results in typical changes in optic disc morphology and visual field. The primary focus in the care of glaucoma patients has been the prevention of ongoing damage to the optic nerve and consequent visual field loss.^3^

Glaucoma, in all its forms, is a major cause of irreversible blindness throughout the world.^4^ It affects more than 13.5 million people over the age of 40 years, with more than 5.1 million people already blind from this disease.^5^ Out of all these glaucoma cases, 10% will be bilaterally blind from the disease, making glaucoma the second leading cause of blindness.^6^

The irony is that this leading cause of irreversible blindness can largely be brought into control most importantly by timely diagnosis, effective treatment, and constant ongoing monitoring. Although it seems attainable in the developing world, glaucoma has proven itself a difficult adversary. Glaucoma requires long-term drug therapy, strict patient compliance, periodical monitoring of ocular parameters, and a reliable follow-up.

Visual field defects in glaucoma tend to affect the midperipheral visual field first and only later in the disease involve the central vision and then fixation. This pattern of visual field loss in glaucoma has led to the impression that the glaucoma patient is asymptomatic until late in the disease. Only when visual field loss impinges upon or involves central vision does the patient become aware of a functional defect.^7^

Objective end points in the management of patients with glaucoma are important and include the level of intraocular pressure (IOP), appearance of the optic nerve head, and status of the visual field. In addition, over the past several years, an increased awareness of the effect of glaucoma on the patient's QoL has developed. Glaucoma may affect a patient's QoL in several ways such as:

- Psychological effects of diagnosis (fear of blindness, fear of affliction of other members of the family, anxiety, depression, etc.),
- Visual effects of glaucoma (decreased visual field and ultimately visual acuity),
- Potential side effects of treatment (medical and surgical), and
- Financial effects (cost of visits and therapy, loss of income because of absenteeism from work).

In opting for treatment, the patient has chosen to endure the side effects of treatment as a trade-off for avoidance of blindness from the disease. Even patients in the early stages of glaucoma experience deficits in QoL associated with self-perceived visual dysfunction. Therefore, maintaining a patient's QoL has always been an important goal for glaucoma treatment.

Physicians have long strived to quantify QoL in patients with glaucoma.^8^ Self-perceived vision-related QoL, however, is not readily quantified by the clinicians without the use of standardized assessment tools. In particular, patients with early or mild glaucoma who exhibit little clinical evidence of disease have been viewed as asymptomatic with regards to vision-related dysfunction. This traditional notion has been fostered by the insidious and often “silent” nature of progressive glaucoma.^9^

When selecting a QoL scale for a glaucoma patient, one might hope the instrument fulfils the following criteria:

- Ease of use in a clinical setting
- Contains minimal complex mathematics
- Allows reproducible data to be obtained
- Correct underlying principles pertaining to glaucoma
- Simple understandable questions with unambiguous answers

Several instruments have been developed for the measurement of vision-related QoL.

These include:

Generic instruments, i.e., not disease state-specific:

- Medical outcomes study short form-36 (SF-36)^10^
- The sickness impact profile (SIP)^11^

Vision-specific instruments:

- Visual Function Questionnaire-14 (VFQ-14)^12^
- The National Eye Institute Visual Function Questionnaire (NEI-VFQ)^13^
- The 25-Item National Eye Institute Visual Function Questionnaire (NEI-VFQ25)^14^

Glaucoma-specific instruments:

- The Glaucoma Symptom Scale (GSS)^15^
- The Comparison of Ophthalmic Medication for Tolerability (COMTOL) Scale^16^
- The Glaucoma Quality of Life-15 (GQL-15)^17^
- The Symptom Impact Glaucoma Score (SIG) and Glaucoma Health Perceptions Index (GHPI)^18^

The glaucoma-specific instruments act as a greater discriminator between glaucoma patients and controls. There appears to be a stronger relationship with objective (clinical) measures of the disease state than the generic instruments.^8^

The GQL-15 (Glaucoma Quality of Life-15) questionnaire is composed of 15 items, 4 domains which address factors of visual disability:

- Central and near vision
- Peripheral vision
- Dark adaptation and glare
- Outdoor mobility^17^

It is short and easy to use. The instrument is based on the premise that perceived visual disability is significantly associated with binocular visual field loss.^8^

Compared to the generic patient-reported outcome measures (PROMs), such as the National Eye Institute Visual Function Questionnaire 25 items, glaucoma-specific questionnaires attached more importance to patients’ visual field loss. The Glaucoma Quality of Life-15 (GQL-15) is one of them and has been proved to perform well among glaucoma patients. Various studies have consistently demonstrated that the GQL-15 score has a strong correlation with objective visual measures.

As this instrument is short and easy to use, it is accepted worldwide. Also when the QoL instrument attempts to glean too much information, they have a tendency to become less user-friendly. Hence, GQL-15 is probably the most useful and clinically relevant tool.

## MATERIALS AND METHODS

The study was conducted on 50 diagnosed cases of chronic open/closed angle glaucoma in one or both eyes attending the Out Patient Department of Ophthalmology, Guru Gobind Singh Medical College and Hospital, Faridkot, Punjab, India. Patients with any nonglaucomatous condition affecting visual functions such as cataract, retinal pathology, symptomatic or uncontrolled systemic disease, nonglaucomatous optic neuropathy were excluded from the study. Patients who underwent ocular laser or surgery in the previous 3 months were also not considered.

Since the data relied on self-reporting of patients’ disability, to minimize recall bias and other nonclinical influences, objective measures of visual function were included in the study.

Patients underwent various tests of visual function. Visual acuity was measured both for distance (Snellen's visual acuity chart) and near vision. Intraocular pressure was measured by a single observer at the same slit lamp using the Goldmann applanation tonometer. As applanation tonometry measurements are affected by the central corneal thickness (CCT), it was measured by ultrasonic pachymetry.^19^ Fundus examination was done using all major tools available—slit lamp biomicroscopy using Volk's 90 D lens examination and direct and indirect ophthalmoscopy to detect glaucomatous changes. Parrish et al. in their study suggested that visual acuity alone is an inadequate indicator of the degree of visual impairment.^20^ Hence, other aspects of visual function were also assessed.

### Visual Field Assessment

All the patients underwent visual field assessment with an automated Humphrey visual field analyser (HFA) using the central 30-2 SITA (Swedish interactive threshold algorithm) strategy. Since binocular field is a more important aspect in patients’ lives, it was tested using Esterman binocular visual field test on the HFA.

For the purpose of statistical analysis, the central visual fields were classified according to severity into 3 groups:^21^

- Mild (unilateral loss with less than half of the visual field lost),
- Moderate (unilateral loss with more than half of the visual field lost, or bilateral loss with less than half of the visual field lost in each eye), or
- Severe (bilateral loss, more than a half of the visual field lost in either eye).

### Stereopsis

It was tested using Randot stereogram test.

### Contrast Sensitivity

It was measured at a distance of 1 m, using the Pelli–Robson contrast sensitivity chart.

### The Questionnaire

As Philipin in his study stated that GQL-15 is more specific and user-friendly than other disease-specific tools,^8^ it was used in this study and was found that patients participated with full cooperation (Table 1). All the above tests were also performed on 50 healthy volunteers for a better comparative evaluation of the quality measure outcomes. GQL-15 includes 15 items divided between 4 factors pertaining to visual disability: central and near vision, peripheral vision, dark adaptation and glare, and outdoor mobility. The GQL-15 items reflect each factor and are represented by a code between 0 and 5 as follows: 0, abstinence from activity for reasons unrelated to vision; 1, no difficulty; and 5, severe difficulty. The subscale score for each factor is calculated as the average of the sum of the item scores. Higher subscale scores indicate greater difficulty in performing vision-related activities and poorer QoL. Scores of the above-mentioned questionnaire for all patients were compared with each other and statistical analysis was carried out.

**Table 1:** The glaucoma quality of life-15 questionnaire: list of daily activities with the strongest relationship with visual field loss in glaucoma

	*None*	*A little bit*	*Some*	*Quite a lot*	*Severe*	*Do not perform for nonvisual reasons*
Factor 1: central and near vision						
• Reading newspapers/doing near work	1	2	3	4	5	0
• Recognizing faces	1	2	3	4	5	0
Factor 2: peripheral vision						
• Seeing objects coming from side	1	2	3	4	5	0
• Walking on uneven ground	1	2	3	4	5	0
• Judging distance of foot to step/curb	1	2	3	4	5	0
• Walking on steps/stairs	1	2	3	4	5	0
• Bumping into objects	1	2	3	4	5	0
• Tripping over objects	1	2	3	4	5	0
Factor 3: glare and dark adaptation						
• Walking after dark	1	2	3	4	5	0
• Seeing at night	1	2	3	4	5	0
• Adjusting to dim light	1	2	3	4	5	0
• Adjusting to bright light or glare	1	2	3	4	5	0
• Going from light to dark room/*vice versa*	1	2	3	4	5	0
• Finding dropped objects	1	2	3	4	5	0
Factor 4: outdoor mobility						
• Crossing the road	1	2	3	4	5	0

### Ethical Consideration

Clearance was obtained from institute ethics committee. An informed consent was obtained from the patient prior to recruitment. The study did not involve any invasive procedure. All therapeutic decisions were taken by the treating physician and no interference was done. Confidentiality of patients was maintained and patients or his/her relative had the right to opt out of study at any given point of time.

### Statistical Analysis

IBM SPSS statistical software version 21 was used for statistical analysis. 95% CI was taken and the *p* value obtained by an independent *t* test and ANOVA test.

## RESULTS

A total of 100 cases were enrolled out of which 50 were diagnosed glaucoma cases and 50 were controls. Almost three-fourth of the glaucoma patients, i.e., 72% were diagnosed as COAG (chronic open angle glaucoma) whereas 24% cases were ACG (angle closure glaucoma). Only 4% cases had normal tension glaucoma (NTG). Based on perimetric evaluation, more than half of the cases, i.e., 60% were diagnosed to have mild glaucoma whereas there was an equal proportion of moderate and severe glaucoma patients, i.e., 20% each in this study. A comparison of the best-corrected visual acuity between various groups of patients was done which showed that patients with mild glaucoma had visual acuity equivalent to controls, i.e., more than 6/18. Whereas, visual acuity in 20% of moderate glaucoma cases ranged from 6/18 to 6/60. Patients with severe glaucoma showed a marked decline in visual acuity in which 40% had visual acuity ≤6/18, 10% had visual acuity ≤6/60, and 20% had visual acuity ≤finger counting at 3 m. The mean binocular contrast sensitivity of controls (2.24 ± 0.06) was better than that of cases included in the study and changed uniformly as we move from mild to moderate and then to severe cases (mean 1.66–0.71). We found in our study that contrast sensitivity did not change much from mild (mean 1.66) to moderate (mean 1.41). However, there was a much higher difference in the contrast sensitivity between moderate (mean 1.41) and severe cases (mean 0.71).

**Fig. 1:**
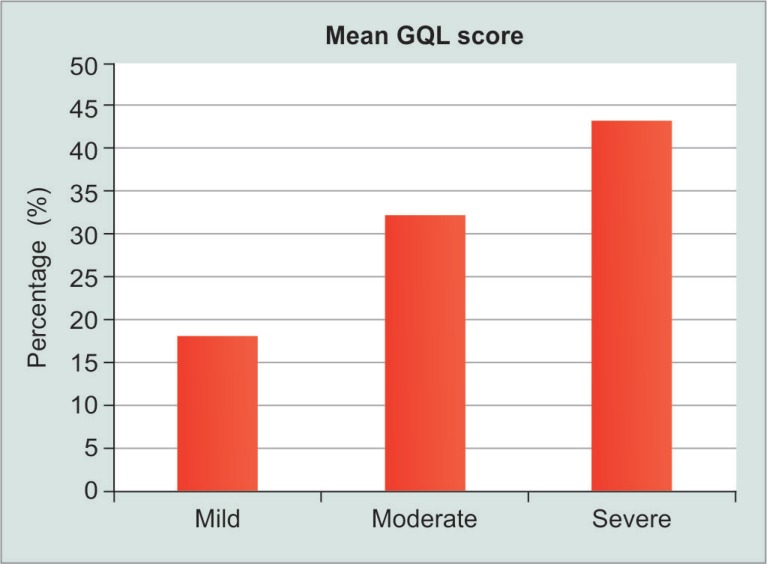
Comparative evaluation of scores of mean GQL-15 questionnaire

The control group had significantly better stereopsis than glaucoma cases with a mean value of 28.50 minutes of arc. Stereopsis in mild cases was 52.16 minutes of arc and in moderate cases it was 117 minutes of arc. Severe cases showed a marked decline in the stereopsis with a mean value of 159 minutes of arc.

### Questionnaire

The mean GQL score of glaucoma cases was 26.00 ± 10.84 and for controls it was 15.02 ± 0.14 (*p* value < 0.05). It was found that mild cases had a mean GQL score of 18.2, moderate had a mean GQL score of 32.2, while patients with severe glaucoma had a mean score of 43.2 (using ANOVA test; *p* value < 0.05). The above findings showed a significant rise in the score between subgroups suggestive of deteriorating visual quality as the severity of glaucoma increased (Fig. 1).

**Fig. 2:**
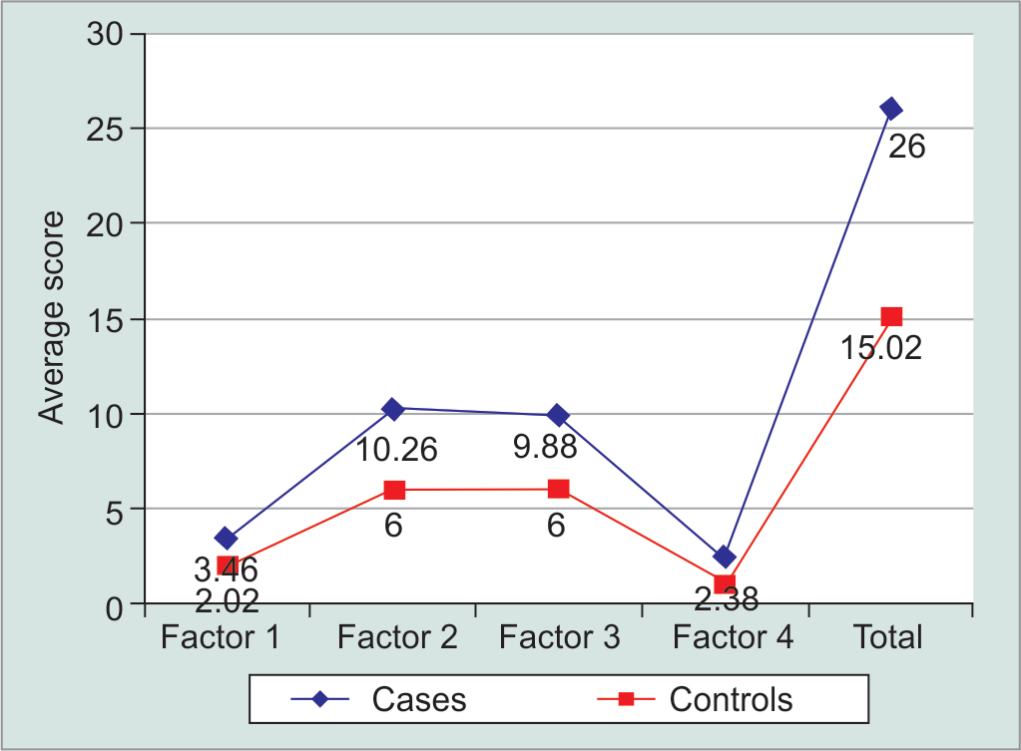
Comparative evaluation of scores of GQL questionnaire between two groups

The GQL questionnaire score was higher for all four domains (1: central and near vision, 2: peripheral vision, 3: dark adaption and glare, and 4: outdoor mobility) in glaucoma patients as compared to the control group (Fig. 2). The results have been summarized in Table 2. Hence, this marked difference in GQL-15 performance measures between patients with glaucoma and healthy individuals suggests a declining vision-related life quality with glaucoma.

**Fig. 3:**
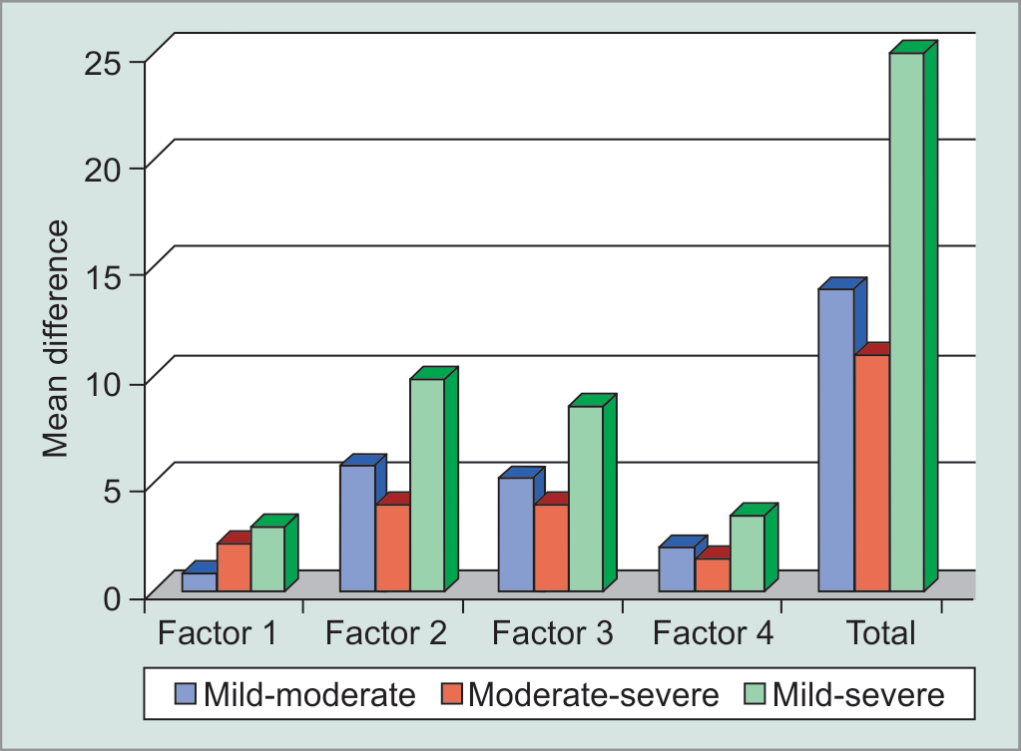
Mean difference in GQL subscale scores in comparison to severity of glaucoma

All the subscale scores also showed a uniform rise in their value as we move from mild to severe cases, thereby, concluding that all the four visual parameters namely central and near vision, peripheral vision, dark adaptation, and outdoor mobility are affected in glaucoma and worsen with an increase in the severity pattern of the disease (Fig. 3).

**Table 2:** Results of the comparison of GQL-15 factor scores between patients with glaucoma and control participants

	*Glaucoma cases*		*Control*		*p value*
	*Mean*	*Standard deviation*	*Mean*	*Standard deviation*	
Factor 1: central and near vision	3.46	1.541	2.02	0.141	0.00
Factor 2: peripheral vision	10.28	4.459	6.00	0.000	0.00
Factor 3: dark adaptation and glare	9.88	4.003	6.00	0.000	0.00
Factor 4: outdoor mobility	2.38	1.589	1.00	0.000	0.00

#### Mild vs Moderate

Factor scores for central and near vision (standardized mean difference (SMD) −0.8, 95% confidence interval (CI) −1.465 to −0.135, *p* value 0.02), dark adaptation and glare (SMD −5.867, 95% CI −7.12 to −4.61, *p* value 0.00), peripheral vision (SMD −5.30, 95% CI −6.457 to −4.143, *p* value 0.02), and outdoor mobility (SMD −2.033, 95% CI −2.542 to −1.525 *p* value < 0.05) differed significantly between patients with mild and moderate glaucoma.

#### Mild vs Severe

Significant differences were observed between factor scores for central and near vision (SMD −3.00, 95% CI −3.743 to −2.257), dark adaptation and glare (SMD −9.867, 95% CI −11.497 to −8.237), peripheral vision (SMD −8.600, 95% CI −9.982 to −7.218), and outdoor mobility (SMD −3.533, 95% CI −3.973 to −3.093). All the factor scores had *p* value < 0.001.

#### Moderate vs Severe

Similar differences were observed on comparing moderate and severe cases of glaucoma, for central and near vision (SMD −2.200, 95% CI −3.384 to −1.016), peripheral vision (SMD −4.000, 95% CI −5.715 to −2.285), dark adaptation and glare (SMD −3.300, 95% CI −5.237 to −1.363), and outdoor mobility (SMD −1.500, 95% CI −2.115 to −0.885). All had *p* value < 0.05 showing significant difference between QoL indices in moderate and severe cases of glaucoma.

As a general rule, it was seen that in all categories of glaucoma, the subscale score difference was highest for peripheral vision meaning thereby, that among all vision-related tasks mentioned in the GQL-15 questionnaire, tasks involving peripheral vision, namely, seeing objects coming from the side, walking on an uneven ground, judging the distance of foot to step or curb, walking on stairs, bumping into objects, and tripping over objects are the ones that are most problematic to the patients in glaucoma over other tasks

In the present study, it was also found that these factors are affected more when we compare patients with mild and moderate glaucoma cases over moderate and severe ones, as the mean difference calculated was significantly higher in the former groups. Exception to this is central and near vision in which difference in scores is higher between moderate and severe glaucoma (difference = 2.2) as compared to mild and moderate ones (difference = 0.8), thereby suggesting that central and near vision is affected more in later stages of the disease.

## DISCUSSION

Quality of life measures are increasingly recognized as important outcomes in understanding the impact of a disease and evaluating the effectiveness of healthcare interventions.^20^ It is important to evaluate the relative importance of symptoms or disabilities to better understand the effect of a disease in a patient's QoL.

Traditionally, the success or failure of medical therapy has been judged by meeting an objective criterion. A consensus has evolved that preferentially values the perception of the patient as the central determinant in monitoring the outcomes of medical intervention.^21,22^ This grows from recognition that patients themselves are not interested in improvements in a biomedical indicator, but rather they are interested in how treatment affects their QoL. Meeting an objective treatment goal, such as lowering of total serum cholesterol levels, remains an important therapeutic concept, but how treatments that achieve this goal affect the perception of well-being and the ability to function effectively as an independent “whole person” is also being considered.^23^ This concept has been utilized in the assessment of chronically ill patients, including those with mental illness, cancer and cardiovascular disease, and in the elderly population. Glaucoma patients infrequently present with visual or systemic symptoms. The failure of primary open-angle glaucoma (POAG) to produce visual symptoms until either visual field loss or diminished central acuity, or both, occurred has thwarted efforts at early diagnosis. Although the objective assessment of progressive glaucomatous damage based on measurement of visual field loss and optic nerve injury is widely accepted, QoL assessments are considerably more complex when the side effects of therapy are superimposed on asymptomatic patients early in the course of their disease. Because both glaucoma and its medical and surgical treatment may affect the global QoL, as well as visually related QoL, assessment of general health status and visual system health status is relevant. Several generic instruments have been developed to measure QoL. Disease-specific instruments, such as the GQL-15 and the glaucoma symptom scale, are specifically designed for use with patients with glaucoma. GQL-15 is a newly developed and validated questionnaire designed to assess QoL in glaucoma patients and focuses on the visual field, which is an important factor in managing glaucoma and has been identified as an indicator of disease severity. Several articles have shown that loss of the visual field exerted a significant impact on QoL in glaucoma patients. For example, a previous study^24^ found that QoL underwent longitudinal changes associated with alterations in the visual field over time in patients with glaucoma. In addition, another study^25^ showed that the progression of loss of sensitivity in the central visual field led to reductions in QoL in patients with glaucoma. The present study sought to examine differences in QoL between patients with mild, moderate, and severe glaucoma.

The GQL-15 questionnaire was developed to evaluate visual field loss specifically. A pilot study began with 62 items that pertained to 10 aspects of daily life; this was later reduced to 15 items that were significantly predictive of visual field loss.^26^ Conjoint analysis showed the relative utility of the GQL-15 questionnaire and the 2 main priorities were identified as central vision (reading or seeing detail) and outdoor mobility (moving around outside the house).^2^ GQL-15 has recently been evaluated using Rasch analysis, and the results showed excellent measurement precision and well-spaced category thresholds.^27^ In addition, the GQL-15 questionnaire outcomes were significantly correlated with visual field loss. Moreover, it is considered easy to understand and can be completed within a reasonable amount of time. The GQL-15 questionnaire was chosen as an assessment tool in the present study, as it has demonstrated good validity, reliability, internal consistency, and reproducibility. However, it focuses mainly on the physical effects of the disease process and does not consider broader QoL-related factors including psychological issues. Kumar et al. compared a general vision-specific instrument, NEIVFQ-25 with two disease-specific instruments, GQL-15, and Viswanathan 10 in patients with varying severity of POAG. All three instruments were reliable in the assessment of mild, moderate, and severe glaucoma. They correlated strongly with each other in most of the related subscales, domains, and questions.^28^

A comparison of patients with and without glaucoma in our study showed that patients with mild, moderate, and severe glaucoma exhibited significantly poorer QoL relative to that observed in patients without glaucoma. The results also indicated that QoL declined in patients with glaucoma who experienced severe loss of the visual field.

These findings were consistent with several studies conducted to evaluate QoL in glaucoma patients such as the study conducted by Goldberg et al. also reported a significantly poorer QoL in glaucoma patients compared with healthy population (*p* < 0.001).^9^ Adeola et al. found that POAG reduces QoL even in very early stages of the disease as there was a significant reduction in the scores.^1^ Arora et al. assessed QoL in newly diagnosed glaucoma patients and compared it to age-matched healthy controls. Glaucoma patients had significantly worse QoL as compared to controls at baseline (*p* < 0.001). 3 months after the initiation of treatment, the overall QoL life significantly worsened from baseline with a decrease in general functioning (*p* < 0.001) and psychosocial impact (*p* = 0.041). In addition, the use of >2 topical medications significantly corelated to poor QoL at 3 months (*p* = 0.01).^29^

Chigozie et al. also found that patients had the greatest difficulty with activities affected by glare and dark adaptation in the GQL-15 questionnaire. While in other questionnaires, driving and general vision were found to be most problematic in the same group of patients.^30^ Vijaya et al. in their study illustrated the challenges faced by glaucoma patients in India. With the help of the GQL-15 questionnaire, they observed that severe vision loss patients faced significant challenges in performing daily tasks and mobility.^31^

The above findings are consistent with the observation that glaucoma affects the midperipheral visual field first and only later in the disease involves the central vision and then fixation. This pattern of visual field loss in glaucoma has led to the impression that the glaucoma patient is asymptomatic until late in the disease. Only when visual field loss impinges upon or involves central vision does the patient become aware of a functional defect.^7^

Hence, this study has clearly proven that the vision-related QoL has to be dealt in all patients of glaucoma. Activities involving the peripheral field are most problematic to the patients. Therefore, the overall management not only includes treating the condition but helping the patients improve their QoL as a whole which can be judged by the various questionnaires mentioned previously. However, there were certain limitations to this present study.

First, classification of glaucomatous patients into mild, moderate, and severe did not distinguish between the peripheral and the central field. Also, there may be some overlap between the three. It also did not assess monocular vs binocular field loss.

Second, we did not consider the prevalence of eyes with prior trabeculectomy which may potentially confound visual function and vision-related QoL in patients with glaucoma. CIGTS reported that compared with patients who received initial medical treatment, patients who received initial surgical treatment had significant (*p* < 0.005) local eye symptoms.^32^

While there is abundance of literature on QoL assessment in glaucoma patients, methods for enhancing it are mentioned less. Educating the patient about the disease and encouraging two-way communication between the patient and the doctor is known to reduce anxiety and stress. Wang et al. suggested improving resilience and social support to improve QoL in glaucoma patients.^33^ Complementary forms of treatment like yoga and meditation have been shown to reduce the associated stress, improve QoL, and also lower IOP in patients with glaucoma. Dada et al.^34^ demonstrated that after a 21-day mindfulness meditation practice, a significant reduction in IOP, stress bio-markers like cortisol, and Interleukin-6, and a significant elevation in beta endorphin, brain-derived neurotrophic factor levels were observed. These results correlated well with gene-expression profiling and improvement in QoL scores.

Gagrani et al. also published similar results after 6 weeks of mindfulness meditation program in POAG patients. In addition, they also demonstrated significant improvement in oxygenated hemoglobin change in the prefrontal cortex using functional near-infrared spectroscopy.^35^

## CONCLUSION

To conclude, glaucoma significantly reduces QoL in patients and every effort should be made to preserve visual function in them. QoL assessments help treating physicians to better appreciate the impact of a disease from the patient's own point of view and suggest priority areas in the individual management plan of patients.

## CLINICAL SIGNIFICANCE

This study paves the way for further research in complementary treatments like meditation to enhance QoL and reduce psychological and physiological stress associated with this chronic disease.
